# *Verticillium dahliae*’s Isochorismatase Hydrolase Is a Virulence Factor That Contributes to Interference With Potato’s Salicylate and Jasmonate Defense Signaling

**DOI:** 10.3389/fpls.2017.00399

**Published:** 2017-03-28

**Authors:** Xiaohan Zhu, Atta Soliman, Md. R. Islam, Lorne R. Adam, Fouad Daayf

**Affiliations:** ^1^Department of Plant Science, University of Manitoba, WinnipegMB, Canada; ^2^Department of Genetics, Faculty of Agriculture, University of TantaTanta, Egypt; ^3^Department of Plant Pathology, Bangladesh Agricultural UniversityMymensingh, Bangladesh

**Keywords:** pathogenesis, *ICSH1*, salicylic acid, jasmonic acid, isochorismatase, virulence factor

## Abstract

This study aimed to dissect the function of the Isochorismatase Hydrolase (*ICSH1*) gene in *Verticillium dahliae*’s pathogenesis on potato. *VdICSH1* was up-regulated in *V. dahliae* after induction with extracts from potato tissues. Its expression increased more in response to root extracts than to leaf and stem extracts. However, such expression in response to root extracts was not significantly different in the highly and weakly aggressive isolates tested. During infection of detached potato leaves, *VdICSH1* expression increased significantly in the highly aggressive isolate compared to the weakly aggressive one. We generated *icsh1* mutants from a highly aggressive isolate of *V. dahliae* and compared their pathogenicity with that of the original wild type strain. The analysis showed that this gene is required for full virulence of *V. dahliae* on potatoes. When we previously found differential accumulation of ICSH1 protein in favor of the highly aggressive isolate, as opposed to the weakly aggressive one, we had hypothesized that ICSH would interfere with the host’s defense SA-based signaling. Here, we measured the accumulation of both salicylic acid (SA) and jasmonic acid (JA) in potato plants inoculated with an *icsh1* mutant in comparison with the wild type strain. The higher accumulation of bound SA in the leaves in response to the *icsh1* mutant compared to the wild type confirms the hypothesis that ICSH1 interferes with SA. However, the different trends in SA and JA accumulation in potato in the roots and in the stems at the early infection stages compared to the leaves at later stages indicate that they are both associated to potato defenses against *V. dahliae*. The expression of members of the isochorismatase family in the *icsh1* mutants compensate that of ICSH1 transcripts, but this compensation disappears in presence of the potato leaf extracts. This study indicates ICSH1’s involvement in *V. dahliae*’s pathogenicity and provides more insight into its alteration of the SA/JA defense signaling’s networking.

## Introduction

*Verticillium dahliae* Kleb., a soil-borne hemi-biotrophic pathogen, causes wilt in more than 200 dicotyledonous plant species and is considered the primary causal agent of the potato early dying (PED) syndrome (Rowe and Powelson, 2002; Johnson and Dung, 2010). The symptoms of Verticillium wilt include chlorosis and necrosis, starting in the lower leaves, as well as vascular discoloration, stunting, and wilting of the infected plant (Pegg and Brady, 2002). Ultimately, *V. dahliae* produces resting structures (microsclerotia) in the necrotic areas of infected tissues, which can sustain germination viability for long period of time up to 10 years or more in soil (Klosterman et al., 2009). When exposed to root exudate, the microsclerotia germinate and the mycelia penetrate the root epidermic cells, and then produce infectious hyphae to colonize the roots (Klosterman et al., 2009). The survival ability of microsclerotia and the wide host range of *V. dahliae* make management strategies for this wilt costly and inefficient, i.e., traditional cultural practices such as crop rotation do not provide useful solutions. Fungicides are ineffective since the infection and colonization occur in the plant’s roots and vascular system before they move upward to the aerial plant parts (Daayf, 2015). Breeding of resistant lines would be paramount but has not been successful. In tomato, resistance to *V. dahliae*, mediated by the *Ve1* gene, has been reported, but is limited to race 1 (Kawchuk et al., 2001; Fradin et al., 2009). In potato (*Solanum tuberosum*), no similar genes have been described so far. However, other studies reported potato defense genes induced in response to this infection (Derksen et al., 2013a).

Plant defense against *V. dahliae* can also be induced using biological means such as non-pathogenic fungi (Veloso and Díaz, 2012), bacteria (Uppal et al., 2008), or plant extracts (El Hadrami et al., 2011). Exploring other new and more efficient Verticillium wilt management strategies require better understanding of the host-*V. dahliae* interaction, i.e., mechanisms of virulence, defense, and how pathogen effector signaling interferes with the plant’s ability to overcome diseases.

Several genes have been studied in relation to their role in *V. dahliae*’s pathogenicity on different plant species. However, little is known about their mechanisms of interference with host defenses (El Hadrami et al., 2011, 2015). Several pathogens have been reported to interfere with their host’s defense signaling, i.e., salicylic acid (SA), and jasmonic acid (JA), in order to successfully invade their tissues (El Oirdi et al., 2011). Potato plants have a high level of endogenous SA, particularly in the leaves (Yu et al., 1997; Navarre and Mayo, 2004). This phytohormone is essential for systemic acquired resistance (SAR) in many species, which makes it a prominent inducible defense hormone, especially against biotrophic pathogens (Yu et al., 1997; Koornneef and Pieterse, 2008). In plants, the phenylpropanoid and isochorismate pathways are two known pathways for SA synthesis (Coquoz et al., 1998; Wildermuth et al., 2001; Pasqualini et al., 2003). SA from the first pathway is apparently required for cell death in the infection site (Pasqualini et al., 2003), while the one from the second pathway seems to be required for SAR responses (Wildermuth et al., 2001). The JA pathway is known to generally interfere with the SA-defense pathway (Derksen et al., 2013b), and was reported to induce systemic tolerance to necrotrophic pathogens (Glazebrook, 1999; Kunkel and Brooks, 2002). The antagonistic effect between SA and JA has been reported in different studies, as part of more efficient defense processes (Pieterse et al., 2009; Davies, 2010). Exogenous SA application improved cotton’s resistance of *V. dahliae* and its toxins (Li et al., 2003; Zhen and Li, 2004; Mo et al., 2016), and of *Arabidopsis* to *V. dahliae* toxins (Jiang et al., 2005). Exogenous methyl jasmonate (MeJA), an analog of JA, increased resistance to *V. longisporum* in both wild type and R-gene-signaling-deficient mutant (*ndr1-1*) lines of *Arabidopsis* (Johansson et al., 2006). Another study reported that jasmonate-deficient tomato mutants (*def1*) were more susceptible to *V. dahliae* compared to the wild type control (Thaler et al., 2004). In *Arabidopsis* mutants, such as ET-signaling-deficient mutants (ein2-1, ein4-1, ein6-1), and JA-signaling-deficient mutants (esa1-1, and pad1-1), but not SA-signaling-deficient mutants (npr1-1, eds1-1, pad4-1, sid2-1) had enhanced susceptibility to *V. longisporum* (Johansson et al., 2006). Genes involved in the SA-signaling pathway such as PAL1, PAL2, and PR-1 had a higher expression in the roots of the moderately resistant than the susceptible potato cultivar in response to *V. dahliae*. On the other hand, PR-2 was up regulated in the leaves of the susceptible potato cultivar (Derksen et al., 2013a). Moreover JA pathway-related genes (PR-3, PR-9, WIN2 and POTLX3) were up-regulated in a susceptible potato cultivar in response to *V. dahliae* (Derksen, 2011; Derksen et al., 2013a), which strongly suggested a partial involvement of SA in potato defense against *V. dahliae* (Derksen et al., 2013a).

The isochorismatase hydrolase (*ICSH1*) (VDAG_05103) of *V. dahliae* belongs to the isochorismatase family. In different organisms, this enzyme catalyzes the conversion of isochorismate into other components, such as 2,3-dihydroxybenzoate and pyruvate (Wildermuth et al., 2001; Soanes et al., 2008). As isochorismate is a very important precursor for SA biosynthesis in plants (Pasqualini et al., 2003), we had hypothesized the potential involvement of isochorismatase in *V. dahliae*’s virulence (El-Bebany et al., 2010), after it was only detected in the proteome of the highly aggressive *V. dahliae* isolate, but not in the weakly aggressive one (El-Bebany et al., 2010). Interestingly, proteins containing an isochorismatase motif have been found in the secretome of five kinds of phytopathogens, but were absent in the secretome of non-pathogenic filamentous ascomycetes (Soanes et al., 2008). Different members of the isochorismatase family were also identified in bacteria, leading the conversion of different substrates into different endpoint compounds (Gehring et al., 1997; Parsons et al., 2003; Künzler et al., 2005; Maruyama and Hamano, 2009), i.e., a bacterial streptothricin hydrolase (SttH) has been identified as a member of the isochorismatase-like hydrolase (ILH) super family, with a primary role in streptothricin (ST)-resistance. The authors had suggested that SttH may have a function in molybdopterin modification (Maruyama and Hamano, 2009). An EntB in *Escherichia coli* has also been described as a isochorismate lyase involved in the enterobactin biosynthetic pathway through utilization of chorismate for the production of 2,3-dihydroxybenzoate (2,3-DHB) (Gehring et al., 1997). In *Pseudomonas aeruginosa*, PhzD was identified as an isochorismatase using 2-amino-2-deoxyisochorismate as a substrate, participating in the phenazine biosynthesis (Parsons et al., 2003). PhzD can also hydrolyze chorismate, vinyl ethers isochorismate, and 4-amino-4-deoxychorismate (Parsons et al., 2003). PchB, which possesses isochorismate pyruvate lyase (IPL) and chorismate mutase (CM) activities in *P. aeruginosa*, catalyzes the conversion of isochorismate into salicylate and pyruvate, and the rearrangement of chorismate into prephenate (Künzler et al., 2005).

Based on the proteomic analysis that showed isochorismatase hydrolase accumulation in the highly aggressive *V. dahliae* isolate Vd1396-9 but not in the weakly aggressive isolate Vs06-14 (El-Bebany et al., 2010), the differentially expressed genes in *V. dahliae*-potatoes interaction, and that both JA and SA signaling pathways seemed to be involved in potato defenses against *V. dahliae* (Derksen, 2011; El-Bebany et al., 2011; Derksen et al., 2013a; El Hadrami et al., 2015), we hypothesized that SA and isochorismatase hydrolase (*ICSH*1) are important components that partially mediate the interaction of *V. dahliae* with potato and other hosts. Indeed, further knocking out of the isochorismatase hydrolase gene in a *V. dahliae* isolate from cotton resulted in attenuating the aggressiveness of the pathogen on this plant species while it accumulated higher levels of SA (Liu et al., 2014). Our hypothesis was that the high level of isochorismatase hydrolase activity in highly aggressive isolates may lead to hijacking the SA pathway in potato plants by hydrolyzing the isochorismate and may represent one of the reasons for higher aggressiveness in certain isolates. Given that previous studies reported that both SA and JA signaling pathways were activated in potato’s response to *V. dahliae* (Derksen, 2011; Derksen et al., 2013a), we speculated that Isochorismatase hydrolase may have differential activities in different isolates, and may play different roles in response to different hosts.

In the present study, our objectives were to: (i) compare the expression of *VdICSH1* in isolates with different pathogenicity levels on potato under different treatments/conditions; (ii) perform a functional analysis of this gene by determining its effect on SA and JA accumulation in potato tissues, and thereby its role in *V. dahliae*’s pathogenicity on potato and selected alternative hosts.

## Materials and Methods

### *V. dahliae* Isolates

*Verticillium dahliae* isolates Vd1396-9 and Vs06-07, from Dr. Daayf’s lab collection, were grown on potato dextrose agar (PDA) media at 24°C for 2 weeks. *V. dahliae* Vd1396-9 and Vs06-07 have been characterized as highly and weakly aggressive isolates, respectively (Uppal et al., 2007; Alkher et al., 2009). The conidial spores were harvested by flooding culture plates with sterilized water, then the concentration was adjusted for each experiment.

### *Agrobacterium*-Mediated Transformation of *V. dahliae*

The open reading frame (ORF) of *ICSH*1 was amplified from genomic DNA of isolate Vd1396-9 with specific primers flanked with restriction sites of EcoRI and BamHI (**Table 1**). The DNA fragment was cloned into the binary vector pDHT (Mullins et al., 2001) and mutagenized using the EZ::TN transposon system (Epicentre Technologies, Madison, WI, USA). *Agrobacterium*-mediated transformation of *V. dahliae* was conducted according to the method described by Dobinson et al. (2004). The transformants were selected in PDA media containing hygromycin B (50 μg/mL). PCR was performed to confirm gene replacement using *ICSH*1-Up-F upstream the target sequence and *ICSH*1-EcoRI-F (**Table 1**). The positive candidate transformants were selected and the expression of *ICSH1* was confirmed by RT-PCR.

**Table 1:** Primer sequences used and accession numbers.

Primer’s name	Primer sequence	Tm	Accession number
ICSH1-EcoRI-F	GGAATTCATGTCCTCATTCCGCTCCAT	70.17	VDAG_05103
ICSH1-BamHI-R	CGGGATCC CTAGTTGATATCCTTGCT	66.01	VDAG_05103
qRTICSH1-F	ATGTCCTCATTCCGCTCCAT	60.27	VDAG_05103
qRTICSH1-R	CTAGTTGATATCCTTGCT	42.93	VDAG_05103
His3-F	ATGGCTCGCACTAAGCAA	54.8	VDAG_10035
His3-R	TGAAGTCCTGGGCAATCT	52.7	VDAG_10035
ICSH1-Up-F	TTTGGCTGCGAAAGACG	57.99	VDAG_05103
qrtVDAG03530F	AGGCTTTCAAACATCCAAC	52.2	VDAG_03530
qrtVDAG03530R	TTCAATAGCGAGTATGTCAGTT	52.4	VDAG_03530
qrtVDAG06170F	TTTCAACCGGCCCTCATT	58.4	VDAG_06170
qrtVDAG06170R	TGGGTGCCAGTCCTTGGT	58.7	VDAG_06171
qrtVDAG06346F	AACCTCTGCCCCAGCGTC	60.4	VDAG_06346
qrtVDAG06346R	GGATGACCTCGGCCTTGTC	59.8	VDAG_06347
potato-EF-F	GATGGTCAGACCCGTGAACAT	57	AJ536671.1
potato-EF-R	GGGGATTTTGTCAGGGTTGT	55.4	AJ536671.1

### Genomic DNA Extraction and Southern Blot Analysis

*Verticillium dahliae* was grown in CDB liquid media for 10 days, then mycelia were collected and ground in liquid nitrogen. The DNA extraction method followed the protocol of Al-Samarrai and Schmid (2000). Genomic DNA of *V. dahliae* (∼20 μg) was digested by restriction enzyme EcoRV overnight at 37°C, and the digested DNA was separated in a 0.8% agarose gel for 6 h at 70 V. The DNA was transferred to a nylon membrane following the protocol of Maruthachalam et al. (2011). Hybridization and detection were conducted with Amersham AlkPhos Direct Labeling and Detection Systems (GE Healthcare Life Sciences, Mississauga, ON, Canada). The signal was visualized on X-ray film (Kodak, Rochester, NY, USA).

### Growth Rate and Conidiation of Mutants

*Verticillium dahliae* mutants *(icsh1-2-3-1* and *icsh1-2-12-1*) and wild type were grown on PDA for 2 weeks, thereafter colony diameters were measured. Each isolate was replicated on 4 petri-plates. Five 1.2 cm diameter mycelial plugs were randomly chosen from 4 week-old *V. dahliae* PDA plates and placed into 40 mL sterilized water, vortexed well then conidia concentration was determined using a hemocytometer.

### Plant Material

Potato cultivars Ranger Russet, a moderately resistant, and Kennebec, susceptible to Verticillium wilt, as well as the susceptible sunflower Hybrid IS8048 and the susceptible tomato variety Bonny Best were used in this study (Madhosingh, 1996; Alkher et al., 2009). Plants were grown in a soil mix containing sand, soil, and peat moss with 1:1:1 ratio in a growth room with a 16/8 h photoperiod and a 22/18°C temperature regimen.

### *V. dahliae* Gene Expression in Response to Elicitation With Different Potato Tissue Extracts

Conidial suspensions of the *V. dahliae* highly aggressive isolate Vd1396-9, the weakly aggressive isolate Vs06-07 and the *icsh1-2-12-1* mutant were adjusted to a concentration of 10^8^ conidia/mL, and 1 mL of each was placed into 100 mL Czapek-Dox Broth (CDB) liquid media (Difco Laboratories, Sparks, MD, USA), then incubated for 1 week at 23.5 ± 1°C. Potato tissue extracts were prepared according to the protocol of El-Bebany et al. (2011). Briefly, 5 g of root, stem, or leaf tissues from 3-week-old healthy Kennebec potato plants were ground into powder using liquid nitrogen with mortars and pestles. Twenty-five mL of sterilized distilled water was added to the powder, then placed on a shaker at 120 rpm (C2 Platform Shaker, Edison, NJ, USA) for 4 h. The mixtures were centrifuged at 2000 *× g* for 5 min, and the supernatant filtered through 0.45 μm syringe filters (Thermo Fisher Scientific, Wilmington, DE, USA). One milliliter of root, stem and leaf extracts were separately added to 1-week-old cultures in CDB media. The experiment was carried out in three replications for each isolate supplemented with different potato extracts. One milliliter of sterilized distilled water was added to the control. The mycelia were harvested a week after treatment, and immediately ground into a powder using liquid nitrogen with pre-chilled mortar and pestle. The mycelial powder was used for RNA extraction.

### Gene Expression Analysis in Infected Potato Detached Leaves

Healthy leaves were cut from 4-week-old Kennebec potato plants, and then petioles placed into 1 mL conidial suspensions of Vs06-07 or Vd1396-9, with a final concentration of 3 × 10^7^ conidia/mL to ensure quick infection. Sterilized water was used for control plants. A half milliliter of sterilized water was added to the detached leaves every second day. The detached leaves were kept at 24 ± 2°C, with a 16/8 h photoperiod day/night. Four to six leaves all from different potato plants were bulked as one replication, with three replications for each isolate for each time point (1 DAI, 3 DAI, 5 DAI, and 8 DAI). Gene expression was analyzed in all treatments. The leaf tissues were immediately frozen in liquid nitrogen, then ground to a fine powder and used for total RNA extraction.

### RNA Extraction and cDNA Synthesis

Total RNA was extracted from potato tissues (100 mg) following the manufacturer’s protocol of the Omega Fungal RNA kit (Omega Bio-Tek, Inc., Norcross, GA, USA). The quantity and quality of the total RNA were analyzed using a NanoDrop 2000 (Thermo Fisher Scientific, Wilmington, DE, USA). The first DNA strand was synthesized from approximately one microgram of the total RNA using Superscript first strand synthesis kit (Life Technologies, Carlsbad, CA, USA).

### Quantitative Real-Time RT-PCR

Quantitative Real-Time RT-PCR was performed according to the protocol of SsoFast EvaGreen Super mix (Bio-Rad Lab, Philadelphia, PA, USA) using a CFX96 Thermal Cycler (Bio-Rad, Hercules, CA, USA). The expression level of the *ICSH1* transcripts was assessed using specific primer sets (**Table 1**). The Histone H3 gene (VDAG_10035) was used as a reference gene. The data was analyzed using the 2^-ΔΔC^_T_ method (Livak and Schmittgen, 2001).

### Pathogenicity Analysis of *Vdicsh1* Mutants

The potato, sunflower and tomato were sown in small plastic trays containing LA4 soil mix (SunGro Horticulture, Agawam, MA 01001, USA) in a growth room for 3 weeks, then roots were washed, trimmed 1–2 cm from the tip and placed in a conidial suspension. The conidia of *V. dahliae icsh1* mutants and wild type were harvested with sterile water and diluted to a concentration of 10^6^ conidia/mL. Sterile water was used as a control treatment. The inoculated plants were transferred to pots containing a pasteurized mixture of sand and soil (2:1). The plants were grown in the same growth room under temperature and photoperiod of 24/18 ± 2°C and 16/8 h day/night, respectively. The total AUDPC of percentage of infection, disease severity, and plant height were analyzed. The vascular discoloration of the stem cross-sections were rated in the last week according to Alkher et al. (2009). Biological replications for potato cultivars Kennebec and Ranger Russet, sunflower Hybrid IS8048, and tomato variety Bonny Best, were 12, 6, 4, and 6, respectively for each treatment. The experiment for potato cultivar Kennebec, sunflower Hybrid IS8048 and tomato variety Bonny Best were repeated with 3, 2, and 2 times, respectively with similar results.

### Analysis of SA and JA Accumulation in Potato Tissues

Three-week-old plants of the potato cultivar Kennebec were inoculated using the root dipping method described above in one of the three conidial suspensions, including *icsh1* mutant (*icsh1-2-12-1*), wild type strain Vd1396-9, or the sterilized water control. The leaves, stems, and roots were sampled at 4, 9, 14, 21, and 35 DAI with three replications. The same tissues were split and extracted for SA and JA analysis.

The extraction and HPLC-PDA-Fluorescence analysis for SA was performed according to El Hadrami et al. (2015). Briefly, 500 mg of potato tissues were ground into powder with liquid nitrogen, then suspended with 490 μl of 80% methanol and 10 μl of 12.5 μg/ml *O*-anisic acid (Sigma–Aldrich Canada Co.) as an internal standard (Meuwly and Métraux, 1993). The mixture was shaken overnight at 4°C and centrifuged at 5,300 rpm for 5 min., the supernatant was evaporated under N_2_, then re-extracted by adding equal volumes of ethyl acetate twice. The acetate phases were combined into a new tube and dried under N_2_. Finally, extracts were re-suspended in 500 μl pure methanol for free SA analysis. The aqueous phases from the previous step were used for bound SA extraction by hydrolyzing in an equal volume of 8N HCl at 100°C for 2 h followed by re-extraction in ethyl acetate. The samples were re-suspended in 500 μl pure methanol and run for SA on HPLC-PDA- Fluorescence (El Hadrami et al., 2015). Bound SA was determined by the amount of free SA released by hydrolysis.

The extraction and UPLC-MSMS analysis for JA was performed according to Henriquez et al. (2016). Briefly, 500 mg of potato tissues were ground into powder with liquid nitrogen, then suspended with 5 ml of extraction solvent (HCl:2-propanol: H2O in a ratio of 2:1:0.002) and 50 μl of 1 μg/ml ( ± )-9,10-dihydrojasmonic acid DHJA (OlChemIm Ltd. Czech Republic) as an internal standard. They were shaken for 30 min at 4°C and mixed with 7 ml of dichloromethane, shaken again for an additional 30 min followed by centrifugation at 5300 rpm for 5 min. The lower phase was transferred into a new tube, dried under N_2_ on ice, then re-suspended in 500 μl 50% methanol.

### Statistical Analysis

Statistical analyses were performed using PROC MIXED with Statistical Analysis Software (SAS) (SAS Institute, Cary, NC, USA; release 9.1 for Windows). Data were checked for normality with PROC UNIVARIATE and outliers were removed based on residuals comparison to critical values for studentized residuals (Lund, 1975) and the Shapiro–Wilk test for normality. Gene expression data were normalized by log^10^ transformation. Mean values were separated using least squared means and letters assigned by the macro PDMIX800.sas (Saxton, 1998) with α = 0.05.

## Results

### Expression of *ICSH1* in *V. dahliae* Isolates under Induction With Plant Extracts

**FIGURE 1:**
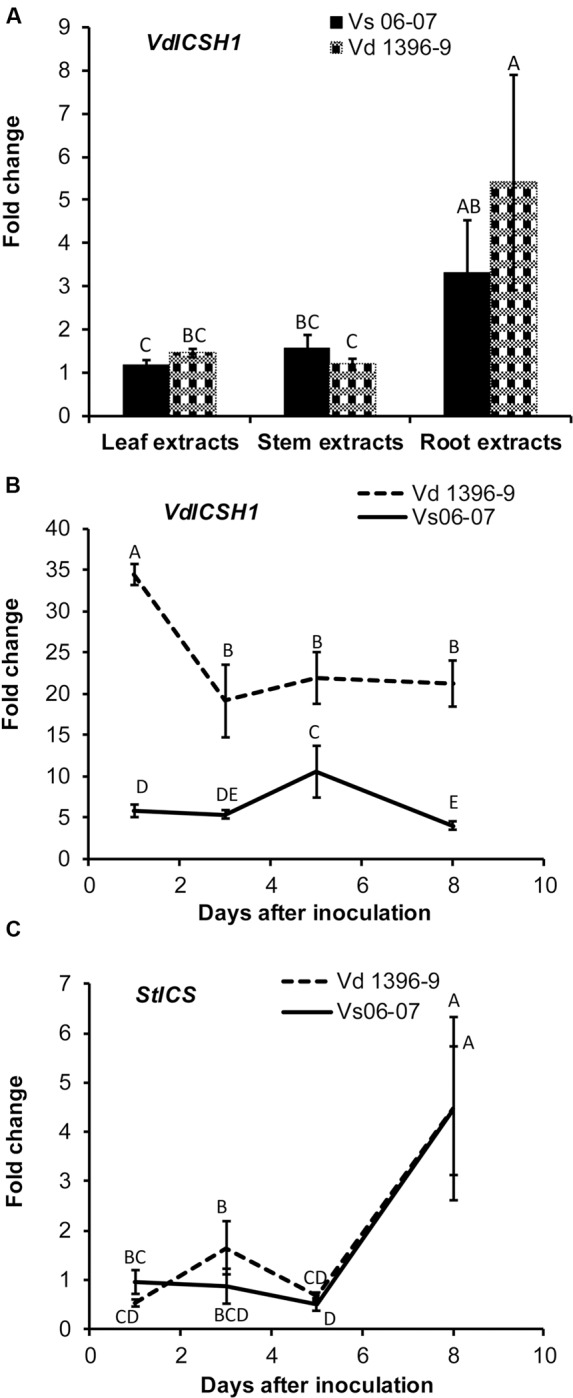
**Expression of *Verticillium dahliae ICSH1* and potato Isochorismate Synthase (*StICS*) under different treatments. (A)** Expression of *Verticillium dahliae ICSH1* under elicitation with different potato tissue extracts. *V. dahliae* highly aggressive isolate Vd1396-9 and the weakly aggressive isolate Vs06-07 were induced in liquid media by different potato tissue extracts. Sterilized distilled water was used as a control treatment. QRT-PCR data were normalized using *V. dahliae* Histone *H3*. The expression data obtained for both isolates under different treatments were analyzed using the 2^-ΔΔC^_T_ method relative to its corresponding isolate cultured in CDB medium with water. The Bars represented by mean values (*n* = 3, with two technical replications) sharing the same letter are not significantly different from each other (*P* < 0.05). **(B)** Expression of *V. dahliae ICSH1* during infection of detached potato leaves. Kennebec potato detached leaves (4-week-old) were placed in conidial suspensions of *V. dahliae* highly aggressive isolate Vd1396-9 and the weakly aggressive isolate Vs06-07; sterilized distilled water was used as a control treatment. Detached leaves from 12 and 18 different individual plants were combined to three biological replicates for each treatment at each time point. QRT-PCR data was *(normalized using *V. dahliae* Histone *H3*. The expression data obtained for both isolates under different treatments were analyzed using the 2^-ΔΔC^_T_ method relative to its corresponding isolate cultured in CDB medium. Point values are represented by mean values (*n* = 3, with two technical replications) sharing the same letter are not significantly different from each other (*P* < 0.05). This experiment was repeated twice. **(C)** Expression analysis of potato Isochorismate Synthase (*StICS*) in response to inoculation with wild type *V. dahliae* isolate. Kennebec potato detached leaves (4-week-old) were placed in conidial suspensions of *V. dahliae* highly aggressive isolate Vd1396-9 and the weakly aggressive isolate Vs06-07; sterilized distilled water was used as a control treatment. Detached leaves from 12 and 18 different individual plants were combined to three biological replicates for each treatment at each time point. QRT-PCR data was normalized using potato elongation factor. The expression data obtained from infected detached leaves under different treatments were analyzed using the 2^-ΔΔC^_T_ method relative to that in uninfected detached leaves. Point values are represented by mean values (*n* = 3, with two technical replications) sharing the same letter are not significantly different from each other (*P* < 0.05).*)

According to previous research, Vd1396-9 is a highly aggressive *V. dahliae* isolate on potato, while Vs06-07 is a weakly aggressive one (Uppal et al., 2007; Alkher et al., 2009). We tested the expression of *ICSH1* in isolates with high and low levels of aggressiveness and under the elicitation of potato tissue extracts. The highly and the weekly aggressive isolates of *V. dahliae* were exposed to potato extracts from different plant parts. The expression of the *ICSH1* increased in response to all extract types, but was significantly higher, in both isolates, in response to the root extract than to the leaf and stem extracts. There were no significant differences between responses of the highly and weekly aggressive isolates (**Figure 1A**).

### Expression of *ICSH1* in *V. dahliae* Isolates after Inoculation of Potato Detached Leaves

The expression level of *ICSH1* was firstly analyzed in the highly and weakly aggressive isolates during the infection of susceptible potato plants (cv. Kennebec), but due to the low biomass of the weakly aggressive isolateVs06-07, it was not detected by QRT-PCR in the plant tissues (Data not show). Therefore, the inoculation was tested with conidial suspensions on detached potato leaves to analyze the expression of *ICSH1* at different time points after inoculation. The gene expression was analyzed for all the treatments, but there was no expression of *ICSH1* on detached leaves without *V. dahliae* infection (**Supplementary Figure S1**). The expression level of *ICSH1* transcripts in inoculated potato leaves followed the same trend with the two *V. dahliae* isolates tested. The expression started by increasing to a high level after 1 day and decreased thereafter. However, a significantly higher expression level was observed with the highly aggressive isolate Vd1396-9, as compared to the weekly aggressive one Vs06-07 (**Figure 1B**).

### Expression of *ICS* in Potato in Response to *V. dahliae*

PAL and ICS are two plant pathways that are both involved in SA synthesis (Wildermuth et al., 2001; Pasqualini et al., 2003). In a previous study, *PAL1* and *PAL2* were shown to be up-regulated in potato in response to *V. dahliae* infection, indicating that PAL-mediated SA signaling pathway is involved in potato plant defense against *V. dahliae* (Derksen et al., 2013a). The expression of a key enzyme of the ICS pathway, Isochorismate synthase (*StICS*), was assessed during inoculation using the two isolates with contrasting levels of aggressiveness. The expression of isochorismate synthase (*StICS*) in potato under *V. dahliae* infection increased significantly 5 DAI, but with no significant difference between the tested isolates (**Figure 1C**).

### *ICSH1* Mutant of the Highly Aggressive *V. dahliae* Isolate Vd1396-9

**FIGURE 2:**
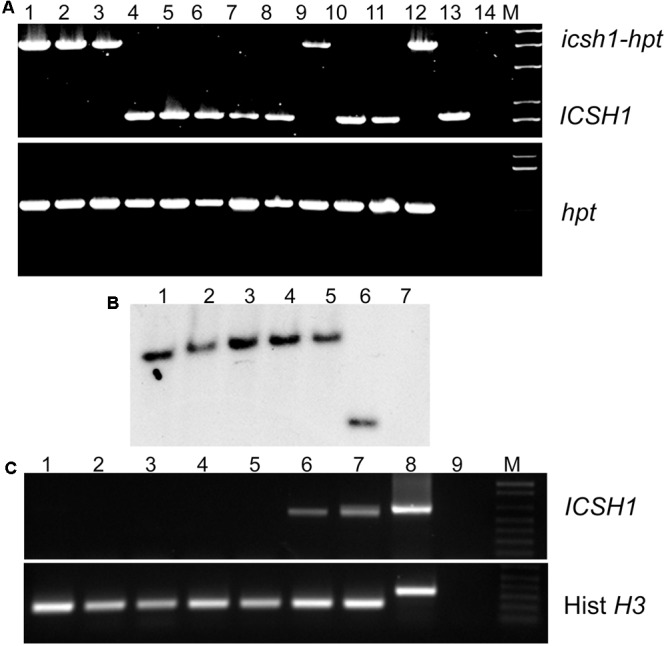
**Identification of *V. dahliae icsh1* mutants. (A)** PCR analysis of transformants of the *icsh1* mutants. Lane 1–12 represent transformants; lane 13, wild type strain Vd1396-9; lane 14, negative control. *hpt*: Hygromycin phosphotransferase gene; *icsh1-hpt:* Isochorismatase hydrolase gene ORF disrupted by insertion of a transposon cassette containing the chloramphenicol resistance gene and the hygromycin phosphotransferase gene. **(B)** Southern blot analysis of the *icsh1* candidate transformants using *hpt* probe. Lane 1–5, positive transformants; lane 6, ectopic control (random insertion without replacing the original *ICSH1* ORF); lane 7, wild type strain Vd1396-9. **(C)** Screening of the transformants of *icsh1* mutant by RT-PCR. Lane 1–5, positive transformants; lane 6, ectopic control; lane 7, wild type strain Vd1396-9; lane 8, DNA of wild type strain Vd1396-9; lane 9, negative control; Hist *H3*: *V. dahliae* Histone *H3*.

To determine the function of ICSH1 in *V. dahliae, Agrobacterium*-mediated transformation was carried out to introduce the transposon insertion into *V. dahliae* Vd1396-9 and disrupt the *ICSH1* as described by Dobinson et al. (2004). A DNA fragment containing the chloramphenicol resistance gene and the hygromycin phosphotransferase gene inserted at the 449th bp of *ICSH1* ORF, was generated in the binary vector pDHt. Five positive *icsh1* mutants were identified by PCR (**Figure 2A**). The integration of the transposon cassette was confirmed by southern blot (**Figure 2B**). On the RNA level, the depletion of *icsh1* transcripts was confirmed by semi-quantitative PCR. The wild type and the ectopic control (*ICSH1-ECT-2-11-3*) (Random insertion in *V. dahliae* genome but without replacing the original *ICSH1* ORF) showed normal levels of *ICSH1* transcripts (**Figure 2C**). Out of the five identified mutants, two (*icsh1-2-3-1* and *icsh1-2-12-1)* were selected randomly for the next steps of phenotype analysis.

### Characterization of the *icsh1* Mutants

**FIGURE 3:**
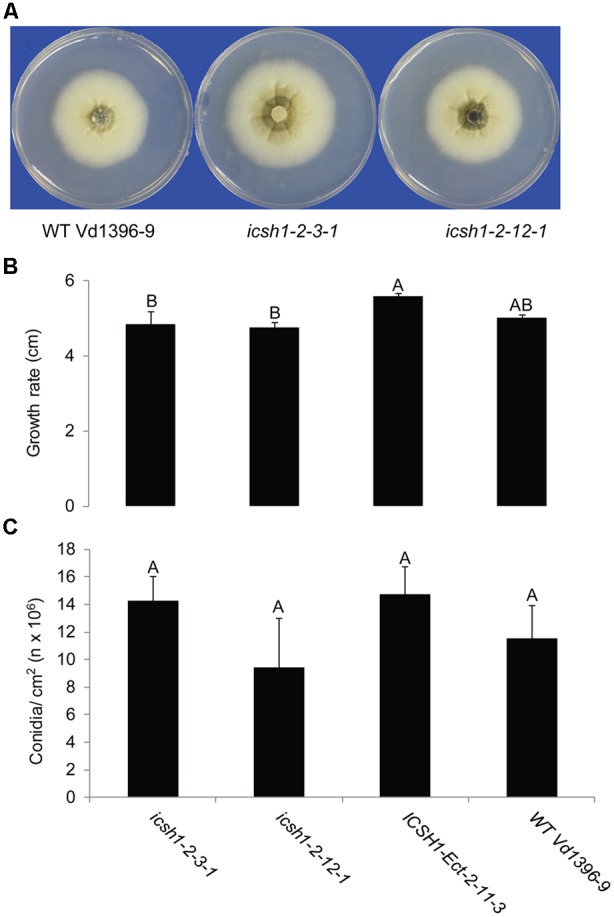
**Morphology, conidiation and grwoth rate of *icsh1* mutants. (A)** The colony phenotype of *icsh1* mutants (*icsh1-2-3-1* and *icsh1-2-12-1*), ectopic control: *ICSH1-Ect-2-11-3* and wild type (Vd1396-9). **(B)** The growth rate of *V. dahliae icsh1* mutants, ectopic control: *ICSH1-Ect-2-11-3* and wild type (Vd1396-9). The growth rates were determined by the colony diameter; **(C)** The conidiation of *icsh1* mutant. The conidia of each isolate were collected from PDA plates, and their concentration determined using a hemocytometer. Bars represented by mean values (*n* = 4) sharing the same letter are not significantly different from each other (*P* < 0.05).

**FIGURE 4:**
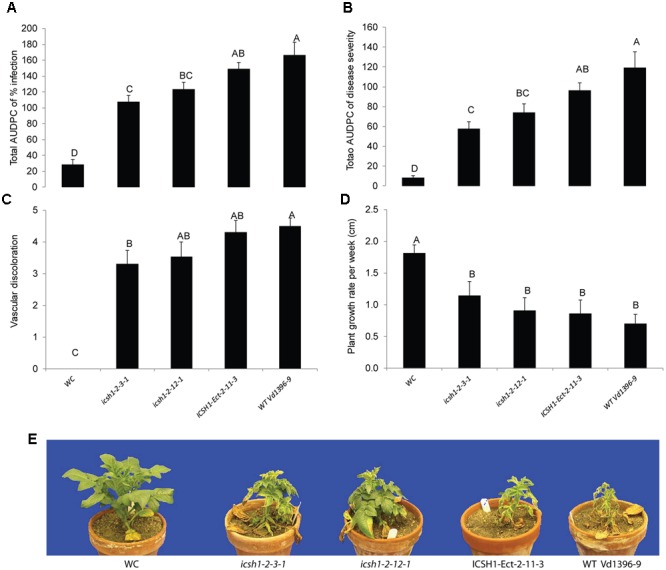
**Pathogenicity of *icsh1* mutants on susceptible potato cultivar (Kennebec).** The percentage of infection, disease severity and plant height were recorded every week. Vascular discoloration of the stem cross-sections was rated at 5 weeks after inoculation with *icsh1-2-3-1* and *icsh1-2-12-1*; ectopic control: *ICSH1-Ect-2-11-3*; wild type: *Vd1396-9;* WC: water control. **(A)** Total AUDPC of percentage of infection; **(B)** Total AUDPC of disease severity; **(C)** Vascular discoloration; **(D)** Growth rate of susceptible potato; **(E)** Kennebec plants infected by *icsh1* mutants at 5 weeks after infection. Bars represented by mean values (*n* = 12) sharing the same letter are not significantly different from each other (*P* < 0.05). This experiment was repeated three times with similar results.

**FIGURE 5:**
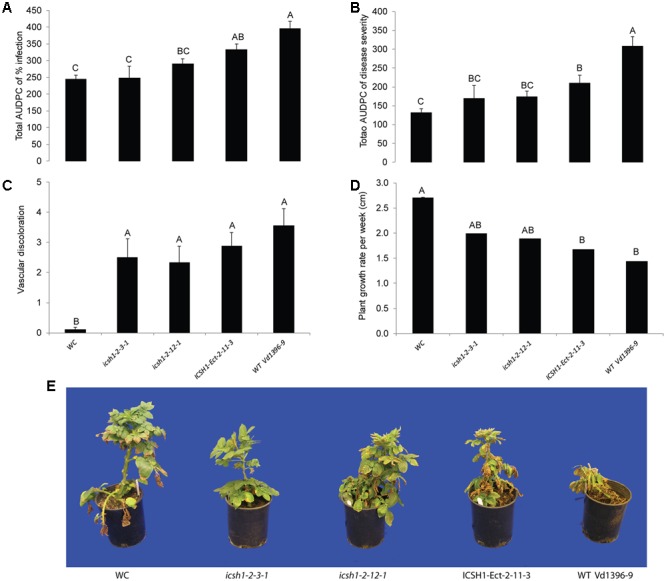
**Pathogenicity of *icsh1* mutants on moderately resistant potato cultivar (Ranger Russet).** The percentage of infection, disease severity and plant height were recorded every week. vascular discoloration of the stem cross-sections was rated at 8 weeks after inoculation with *icsh1-2-3-1* and *icsh1-2-12-1*; ectopic control: *ICSH1-Ect-2-11-3*; wild type: *Vd1396-9;* WC: water control. **(A)** Total AUDPC of percentage of infection; **(B)** Total AUDPC of disease severity; **(C)** Vascular discoloration; **(D)** Growth rate of moderately resistant potato; **(E)** Ranger Russet plants infected by *icsh1* mutants at 8 weeks after infection. Bars represented by mean values (*n* = 6) sharing the same letter are not significantly different from each other (*P* < 0.05).

The phenotype, growth rate, conidiation, microsclerotia formation of *icsh1* mutants were tested on PDA plates, while the pathogenicity was tested on susceptible and moderately resistant potato cultivars, on sunflower and on tomato plants. The morphology, conidiation and microsclerotia formation were not affected in the tested *V. dahliae icsh1-2-3-1* and *icsh1-2-12-1* mutants (**Figure 3**). There was no significant difference among the *icsh1* mutants and the wild type neither in growth rate nor in conidial formation. On the other hand, the total area under disease progress curve (AUDPC) of infection and disease severity showed significant reduction with *V. dahliae* mutants compared to the wild type in both susceptible and moderately resistant potato cultivars (**Figures 4, 5**). The plant height and vascular discoloration followed the same trend in both cultivars, with no significant difference between the mutants and the wild type. In addition to potato, the wild type and *icsh1* mutants were tested on sunflower and tomato plants. In sunflower, both wild type and *icsh1* mutant were able to induce disease, but there were no significant differences between wild type and mutants in the total AUDPC of either infection or disease severity (**Supplementary Figure S2**). In tomato plants, there were no significant differences observed in any of the tested parameters for both pathogen isolates (**Supplementary Figure S3**). In conclusion, the ICSH1 is important for virulence of *V. dahliae* on potato but not on sunflower and tomato.

### Expression of Other Members of the Isochorismatase Family in *V. dahliae* under Induction With Plant Extracts

**FIGURE 6:**
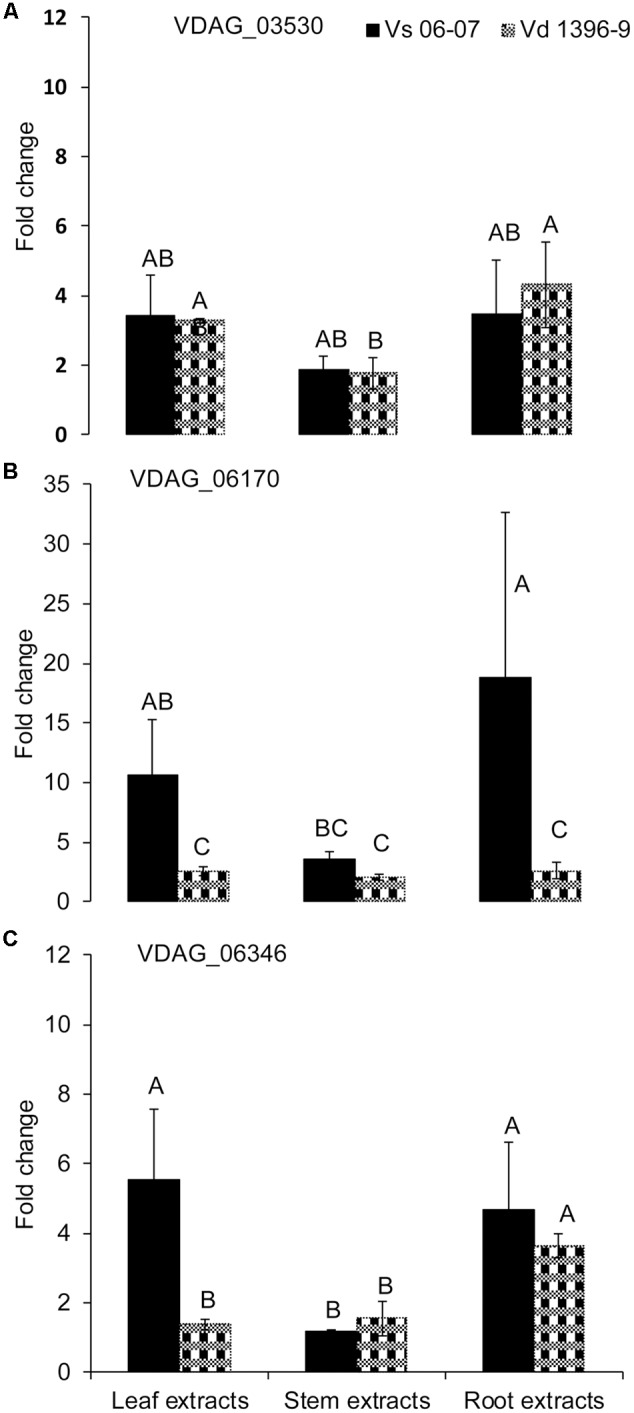
**Expression analysis of isochorismatase family members in *V. dahliae* wild type isolates under elicitation with different potato tissue extracts.** *Verticillium dahliae* highly aggressive isolate Vd1396-9 and weakly aggressive isolate Vs06-07 were elicited in liquid media by different potato tissue extracts. Sterilized distilled water was used as a control treatment. QRT-PCR data was normalized using *V. dahliae* Histone *H3*. **(A)** VDAG_03530; **(B)** VDAG_06170; **(C)** VDAG_06346. Each gene’s expression data obtained from both isolates under different treatments were analyzed using the 2–ΔΔCT method relative to its corresponding isolate cultured in CDB medium with water. Bars represented by mean values (*n* = 3, with two technical replications) sharing the same letter are not significantly different from each other (*P* < 0.05).

To determine potential roles of other genes from the isochorismatase family in presence and absence of *ICSH1*, the expression of five genes from the isochorismatase family in *V. dahliae* (Accession # VDAG_06346, VDAG_03530, VDAG_06170, VDAG_06688, and VDAG_08870) was tested by QRT-PCR in a highly and a weakly aggressive isolates, and in the *isch1* mutant (*icsh1-2-12-1*) under induction with potato extracts. The same volume of water was added in the medium for each strain as a control to potato extracts. All data was analyzed using the 2^-ΔΔC^_T_ method (Livak and Schmittgen, 2001). In both strains, the expression level of VDAG_06688 and VDAG_08870 was too low to be detected. The expression of VDAG_03530 increased in both the highly and weekly aggressive isolates in response to all types of extracts, without significant differences between the isolates (**Figure 6A**). The expression of VDAG_06170 increased in both the highly and weekly aggressive isolates in response to all types of extracts (**Figure 6B**), with a higher level in the weakly aggressive isolate in response to potato leaf and root extracts. In response to potato leaf extracts, the expression of VDAG_06346 increased more in the weakly aggressive isolate than in the highly aggressive one but such increase was similar in the two isolates in response to both stem and root extracts (**Figure 6C**).

**FIGURE 7:**
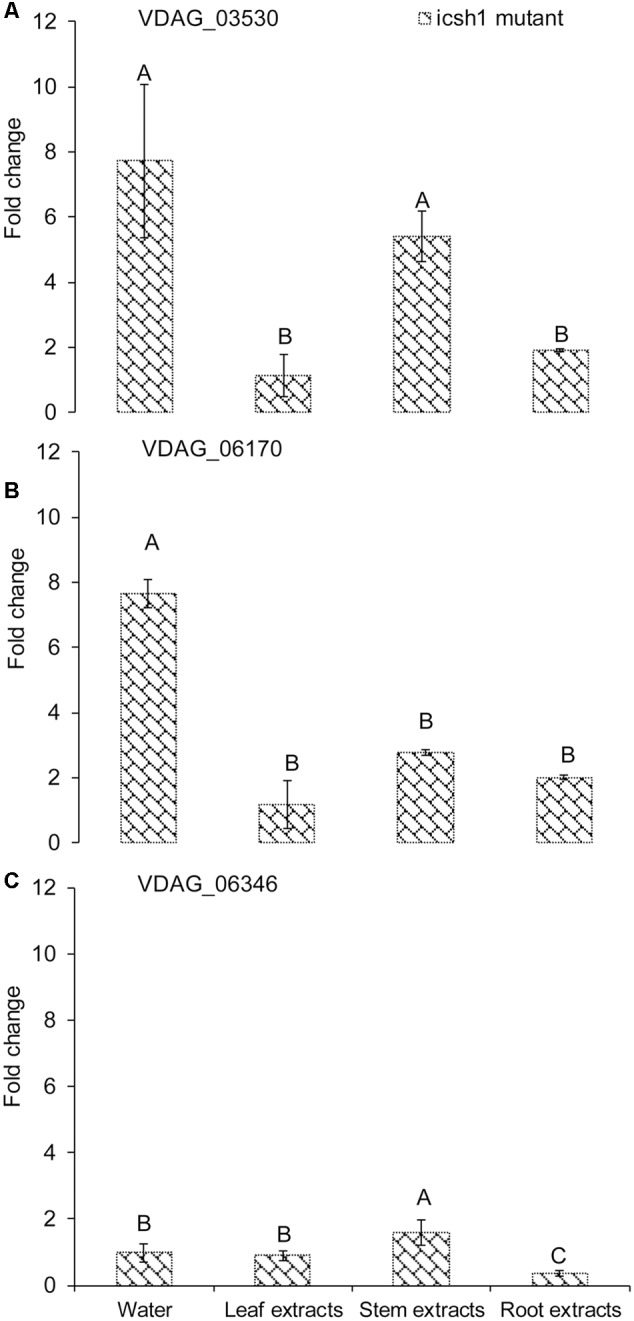
**Expression of isochorismatase family members in *V. dahliae icsh1* mutant under elicitation with different potato tissue extracts.** *Verticillium dahliae* wild type strain Vd1396-9 and mutant *icsh1-2-12-1* were elicited in liquid media by different potato tissue extracts. Sterilized distilled water was used as a control treatment. QRT-PCR data was normalized using *V. dahliae* Histone *H3*. **(A)** VDAG_03530; **(B)** VDAG_06170; **(C)** VDAG_06346. Each gene’s expression data obtained with the *icsh1* mutant in CDB with water control were analyzed using the 2^-ΔΔC^_T_ method relative to that of wild type Vd1396-9 cultured in CDB medium with water. Each gene’s expression data obtained for the *icsh1* mutant under each treatment were analyzed using the 2^-ΔΔC^_T_ method relative to that of wild type Vd1396-9 cultured in CDB medium with the same treatment. Bars represented by mean values (*n* = 3, with two technical replications) sharing the same letter are not significantly different from each other (*P* < 0.05).

Comparison of the expression of the above-cited genes in the *isch1* mutant and the wild type Vd1396-9 under treatments with different extracts revealed the expression levels of VDAG_03530 and VDAG_06170 to be significantly higher in the *isch1* mutant than those in the wild type isolate Vd1396-9 (**Figure 7** and **Supplementary Figure S4**). To make these comparisons more clear, the data were shown in terms of expression of these genes in *icsh1* relative to the wild type strain under each treatment (**Figure 7**). Both stem and root extracts induced a higher expression of VDAG_03530 and VDAG_06170 in the mutant compared with the wild type (**Figures 7A,B**). Moreover, compared with the wild type with no treatment, the expression of VDAG_03530 in *isch1* mutant was about eight-fold higher in response to water (control), stem extracts, and root extracts (**Supplementary Figure S4A**). Similarly, the expression of VDAG_06170 in *isch1* mutant in response to water (control), stem extracts and root extracts was about 7-fold, 5-fold, and 4-fold respectively to that of the wild type with no treatment (**Supplementary Figure S4B**). Inversely, the expression of VDAG_06346 was higher in the wild type isolate compared to the *icsh1* mutant in response to root extract (**Supplementary Figure S4C**).

In conclusion, the expression of VDAG_03530 and VDAG_06170 in the mutants compensate that of ICSH1 transcripts, but this compensation is not apparent in presence of potato leaf extracts (**Figure 7** and **Supplementary Figure S4**).

### SA and JA Quantification in Potato Plants under Infection

To determine the role of ICSH1 in altering the accumulation of SA and possibly JA, the *icsh1* mutant (*icsh1-2-12-1*) and wild type strain Vd1396-9 were selected for this experiment. The susceptible potato cultivar Kennebec was inoculated by root dip in a conidial suspension, while plant roots dipped in sterilized water were used as a control. The plant tissues were sampled at 4, 9, 14, 21, and 35 days after infection. Quantification of SA in different parts of potato plants after inoculation with *V. dahliae* wild type strain and the *icsh*1 mutant was done using HPLC-Fluorescence. SA levels were significantly higher in all infected plant parts with both wild type and the *icsh1* mutant.

**FIGURE 8:**
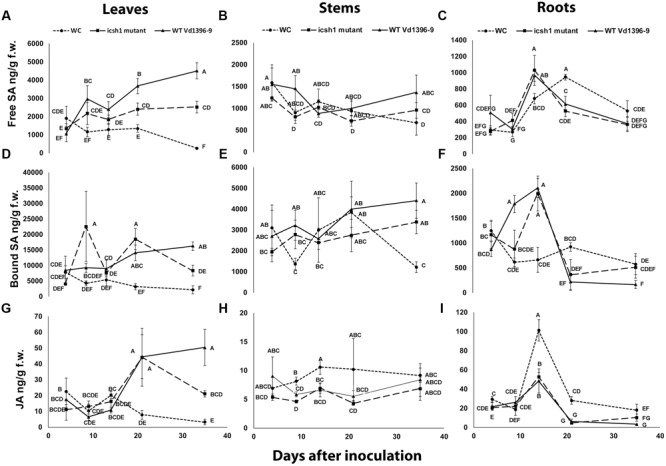
**Quantification of SA and JA concentration in potato during infection.** Susceptible potato cultivar Kennebec was inoculated with conidial suspensions from *icsh1-2-12-1* and wild type strain Vd1396-9; sterilized water used as a control treatment (WC: water control). **(A)** Free SA in leaves; **(B)** Free SA in stems; **(C)** Free SA in roots; **(D)** Bound SA in leaves; **(E)** Bound SA in stems; **(F)** Bound SA in roots; **(G)** JA in leaves; **(H)** JA in stems; **(I)** JA in roots. Point values represented by mean values (*n* = 3) sharing the same letter are not significantly different from each other (*P* < 0.05).

In the roots, the level of free SA in response to inoculation went higher at 14 DAI thereafter decreasing compared to the water control (**Figure 8C**). At early stages of leaf infection (9 DAI), bound SA accumulation was significantly higher in response to the *icsh*1 mutant, compared to the wild type, whereas the opposite happened in the roots (**Figures 8D,F**). In stems, there were no significant differences between the SA accumulation in response to *V. dahliae* inoculation and water control until 35 DAI (**Figures 8B,E**). In leaves, SA levels were significantly higher in response to the wild type, compared to the *icsh*1 mutant, starting from 21 DAI for the free SA and 35 DAI for the bound-SA (**Figures 8A,D**). JA was also quantified in leaf, stem and root tissues of cultivar Kennebec after inoculation with *V. dahliae* wild type and *icsh1* mutant using UPLC-MSMS. There were no significant differences between plants inoculated with the wild type and the mutant in terms of JA induction in the stems and roots, which was less than in the wounded control (**Figures 8H,I**). In the leaves, however, both the mutant and wild type induced more JA than the control at 21 DAI. At 35 DAI JA levels in the wild type were significantly higher than the *icsh1* mutant or water control (**Figure 8G**).

In conclusion, potato accumulated SA in the roots at early infection stages and in the stems in both early and later stages, while the accumulation of SA and JA occurred in the leaves at later stages in response to *V. dahliae* infection. The SA and JA accumulation followed different trends in the roots and stems at the early stages, and similar trends in the leaves at later stages. The *icsh1* mutant induced a high level of bound-SA in the leaves only at 9 DAI, and thereafter decreased. Taken together, the results indicate that the wild type *V. dahliae* isolate induced more free-SA than the mutant in the leaves, and more bound-SA in the roots. However, in absolute value, the total SA induced by the mutant in the leaves, at 9 DAI, is higher than the free SA induced by the wild type in the roots, stems, and leaves, combined (**Figure 8**).

## Discussion

In the current study, *Vdicsh1* mutants of *V. dahliae* were generated and functionally characterized on potato and other host species. As we initially predicted the possible role of this gene as a virulence factor using proteomics studies (El-Bebany et al., 2010), *V. dahliae* ICSH1 was found to reduce SA synthesis in cotton (Liu et al., 2014), and in potato tissues in the present study. However, the intricate complexity of *V. dahliae*’s interaction with its hosts raises many questions regarding the mechanisms, and the spatio-temporal unfolding of the effects of ICSH1 during infection and colonization of the host.

The differential expression of *ICSH1* in highly vs. weakly aggressive *V. dahliae* isolates in culture as well as in detached potato leaves inoculated with this pathogen demonstrated its role as a pathogenicity factor on this host plant. The highest induction of *ICSH1* in response to extracts from the roots, compared to leaves and stems suggests the importance of this gene in establishing infection, since *V. dahliae* is a soilborne pathogen and germination of its microsclerotia is known to be stimulated by root exudates (Klosterman et al., 2009). Potato tissues responded to inoculation by activating the isochorismate synthase (ICS) encoding the key enzyme in the ICS pathway. The expression level of potato ICS was upregulated in the leaves but was not associated with the level of the pathogen aggressiveness (**Figure 1**). Both PAL and ICS pathways are involved in SA synthesis (Wildermuth et al., 2001; Pasqualini et al., 2003). PAL-mediated SA signaling pathway is arguably involved in potato early response to wounding and infection by *V. dahliae*, with *PAL1* expressed higher in susceptible potato Kennebec than in moderately resistant potato Ranger Russet in both leaves and roots and *PAL2* only in the leaves (Derksen et al., 2013a). The activity of ICS, which would also result in SA accumulation, was maintained over time, but was higher 8 DAI (**Figure 1**).

The reduced total AUDPC of percent infection and disease severity as a result of *ICSH1* mutation seems to be associated with the reduction of ICSH1 activity. Similar results were observed in a study on isochorismatase hydrolase (*icsh1*) mutants from *V. dahliae* on cotton and *Phytophthora sojae* on soybean, respectively. Silencing the expression of *V. dahliae VdIcs1* and *P. sojae PsIcs1* resulted in a change in the virulence of these pathogens (Liu et al., 2014). Together, these results demonstrate the importance of ICSH1 as a virulence factor in *Verticillium* and possibly other filamentous fungi. Unlike other virulence factors, ICSH1 protein lacks the transit peptide and the secretion route of this protein is still unknown (Bendtsen et al., 2004; Liu et al., 2014). Proteomic analysis of several phytopathogenic fungi showed the presence of isochorismatase motif proteins in the secretome of phytopathogenic but not in non-pathogenic filamentous ascomycetes (Soanes et al., 2008). Several members of the ILH superfamily were also identified in bacteria; i.e., *Pseudomonas aeruginosa* PchB possesses IPL and CM, which catalyze the conversion of isochorismate into salicylate and pyruvate (Gehring et al., 1997; Parsons et al., 2003; Künzler et al., 2005; Maruyama and Hamano, 2009).

Plants respond to pathogens by various defense mechanisms. In response to *V. dahliae*, SA accumulates using both the PAL (Derksen et al., 2013a) and ICS pathways. The timing difference in *PAL* and *ICS* expression, along with the timing in SA accumulation in the roots vs. leaves is in agreement with the suggestion that the ICS pathway is associated with SAR, while the PAL pathway is essential for initiating cell death in the infection site (Gaffney et al., 1993; Rairdan and Delaney, 2002; Pasqualini et al., 2003; Holuigue et al., 2007). A study on *Arabidopsis* has also shown that jasmonates play a central role in early SAR signaling before systemic SA accumulation (Truman et al., 2007). The JA pathway may also play a role in potato SAR response to *V. dahliae* invasion.

Under *V. dahliae* infection, potato roots accumulated higher amounts of free and bound SA than the water control at early stages, while JA was detected at lower levels in the infected potato roots, compared to wounded non-inoculated control. This indicated that potato roots may respond to *V. dahliae* by accumulating more SA and less JA at early stages of infection. However, it is not clear whether the pathogen is the one suppressing JA accumulation (**Figure 8I**). Potato stems also responded to *V. dahliae* by accumulating more SA and less JA. At later stages, the potato leaves responded to *V. dahliae* infection by accumulating both SA and JA. The antagonistic effect between JA and SA is known (Pieterse et al., 2009; Davies, 2010). If such antagonism occurred in our experiments, that would explain the levels of SA and JA in the potato roots and stems in response to *V. dahliae* at early stages of infection. In potato leaves, at later stages, the response to *V. dahliae* involved both SA and JA accumulation.

Generally, the *icsh1* mutant induced a high level of accumulation of bound-SA in the leaves at 9 DAI. In *Arabidopsis*, the SA is glucosylated by UDP-glucosyltransferases UGT74F1 and UGT74F2 into bound-SA in the cytosol (Lim et al., 2002; Song, 2006; Dean and Delaney, 2008; Dempsey et al., 2011). Bound-SA would then be transported and stored in the vacuole (Dempsey et al., 2011). Bound-SA could be hydrolyzed into SA *in planta*, but it is unclear whether bound-SA is biologically active as such (Dempsey et al., 2011). In tobacco, the hydrolysis of bound-SA into SA by extracellular glucosidases, followed by its injection into tobacco leaves induced the expression of PR-1 (Hennig et al., 1993). The bound-SA in potatoes may also be very important for plant defense, because most fungal plant pathogens are capable of producing hydrolases as part of their pathogenesis processes (Williams and Orpin, 1987; Kawai et al., 2004; Colen et al., 2006; Chen et al., 2008). The increase of bound-SA in the leaves inoculated with the *icsh1* mutant at 9 DAI may also indicate that bound-SA can be an important element for plant defense and needs to be overcome by *V. dahliae* for successful infection. Later, however, SA accumulated at lower levels in potato leaf tissues infected by *icsh1* mutant than those infected by wild type *V. dahliae* strain Vd1396-9. In potato roots, SA accrued in a similar manner in those infected by *V. dahliae* wild type strain Vd1396-9 and those infected by the *icsh1* mutant. This suggested that potato plants infected either by wild type strain or the *icsh1* mutant may compensate the depletion of the isochorismate hydrolyzed by the pathogen’s *ICSH*. This strategy would take place partly by increasing export of chorismate from the plastids into the cytosol (Djamei et al., 2011), where chorismate is converted into isochorismate by ICS (Pasqualini et al., 2003). This may help to retain the cellular homeostasis of isochorismate. This hypothesis also matches the fact that both ICS and PAL are activated during infection, because chorismate is also a precursor of the PAL pathway (Pasqualini et al., 2003). On the other hand, increasing the expression of other genes from isochorismatase family such as VDAG_03530 and VDAG_06170 in the *icsh1* mutant may compensate for the lack of the ICSH1 activity, but this compensation seems inhibited in presence of the potato leaf extracts, and this may also explain that the *icsh1* mutant induced a high level of accumulation of bound-SA in the leaves at 9 DAI. Oppositely, in the *V. dahliae*-cotton and *P. sojae*-soybean pathosystems, SA accumulated at a significantly higher level in the plant roots after infection with *VdIcs1* and *PsIcs1* mutants. This may be due to the lack of activity compensation for the isochorismatase hydrolase, which hydrolyzes the isochorismate and suppresses SA biosynthesis (Liu et al., 2014). In the present study, it is intriguing that the effects of *icsh1* mutation were observed on potato, as the host of origin of the highly aggressive wild type tested strain, but not on sunflower or tomatoes. This calls for more studies on such associations and the level of specificity observed in these responses.

The present study offers a more dissected analysis of the potential roles of ICSH1 in *V. dahliae’s* pathogenesis processes and sheds more light into its effect on the complex potato signaling in response to this important wilt pathogen.

## Author Contributions

XZ planned and run most of the experiments in this manuscript and contributed to the writing of the results; AS provided technical assistance for the molecular work and writing of the manuscript; MI helped with the fungal transformation work, LA provided technical assistance for inoculations and statistical analysis; FD contributed the idea, supervised the work, and contributed to the writing of the manuscript.

## Conflict of Interest Statement

The authors declare that the research was conducted in the absence of any commercial or financial relationships that could be construed as a potential conflict of interest.

## Supplementary Material

FIGURE S1 **Amplification of *V. dahliae ICSH1* in all potato detached leaves’ treatments at 1 DAI.** Kennebec potato detached leaves from 4-week-old plants were placed in conidial suspensions of the *V. dahliae* highly aggressive isolate Vd1396-9, the weakly aggressive isolate Vs06-07, or sterilized distilled water as a control treatment. **(A)** Amplification of *VdICSH1* in all potato detached leaves’ treatments at 1 DAI; lane 1: negative control; lane 2: Vd1396-9 genomic DNA; lane 3: cDNA of Vd1396-9; lane 4: cDNA of Vs06-07; lane 5–7: cDNA of potato detached leaves inoculated with Vd1396-9 at 1 DAI; lane 8–10: cDNA of potato detached leaves inoculated with Vs06-07 at 1 DAI; lane 11–13: cDNA of water control potato detached leaves at 1 DAI; M: DNA marker. **(B)** Amplification of potato elongation factor (EF) in all potato detached leaves’ treatments at 1 DAI; lane 1: negative control; lane 2: cDNA of Vd1396-9; lane 3: cDNA of Vs06-07; lane 4–6: cDNA of potato detached leaves inoculated with Vd1396-9 at 1 DAI; lane 7–9: cDNA of potato detached leaves inoculated with Vs06-07 at 1 DAI; lane 10–12: cDNA of water control potato detached leaves at 1 DAI; M: DNA marker.

FIGURE S2 **Pathogenicity of *icsh1* mutant on susceptible sunflower line IS8048.** Roots of the susceptible sunflower line IS8048 were washed and placed in a conidial suspension of *V. dahliae*. The percentage of infection, disease severity and plant height were recorded every week. The vascular discoloration of the stem cross-sections were rated at 8 weeks after inoculation, with *icsh1-2-3-1* or *icsh1-2-12-1*; ectopic control: *ICSH1-Ect-2-11-3*; wild type: *Vd1396-9*; WC: water control. **(A)** Total AUDPC of percentage of infection; **(B)** Total AUDPC of disease severity; **(C)** Vascular discoloration; **(D)** Growth rate of susceptible sunflower; **(E)** Sunflower line IS8048 infected by *icsh1* mutants at 8 weeks after infection. Bars represented by mean values (*n* = 4) sharing the same letter are not significantly different from each other (*P* < 0.05). This experiment was repeated twice.

FIGURE S3 **Pathogenicity of the *icsh1* mutant on susceptible tomato variety Bonny Best.** Roots of the susceptible tomato variety Bonny Best, were washed and placed in a conidial suspension of *V. dahliae*. The percentage of infection, disease severity and plant height were recorded for each week after inoculation with *icsh1-2-3-1* or *icsh1-2-12-1*; ectopic control: *ICSH1-Ect-2-11-3*; wild type: *Vd1396-9*; WC: water control. **(A)** Total AUDPC of percentage of infection; **(B)** Total AUDPC of disease severity; **(C)** Growth rate of susceptible tomato; **(D)** Tomato variety Bonny Best infected by *icsh1* mutants at 8 weeks after infection. Bars represented by mean values (*n* = 6) sharing the same letter are not significantly different from each other (*P* < 0.05). This experiment was repeated twice.

FIGURE S4 **Expression of isochorismatase family members in the *V. dahliae icsh1* mutant and wild type strain Vd1396-9 under elicitation with different potato tissue extracts.** *Verticillium dahliae* wild type strain Vd1396-9 and *icsh1-2-12-1* were induced in liquid media by different potato tissue extracts. Sterilized distilled water was used as a control treatment. QRT-PCR data was normalized using *V. dahliae* Histone *H3*. **(A)** VDAG_03530; **(B)** VDAG_06170; **(C)** VDAG_06346. Each gene’s expression data obtained from the *icsh1* mutant and wild type strain Vd1396-9 under all treatments and control were analyzed using the 2^-ΔΔC^_T_ method relative to that of wild type Vd1396-9 cultured in CDB medium with water. Bars represented by mean values (*n* = 3, with two technical replications) sharing the same letter are not significantly different from each other (*P* < 0.05).
