# Supplementary figures and images for: Verticillium dahliae’s Isochorismatase Hydrolase Is a Virulence Factor That Contributes to Interference With Potato’s Salicylate and Jasmonate Defense Signaling
Source: Front Plant Sci. 2017 Mar 28;8:399. doi: 10.3389/fpls.2017.00399 (PMC5368275; doi:10.3389/fpls.2017.00399)

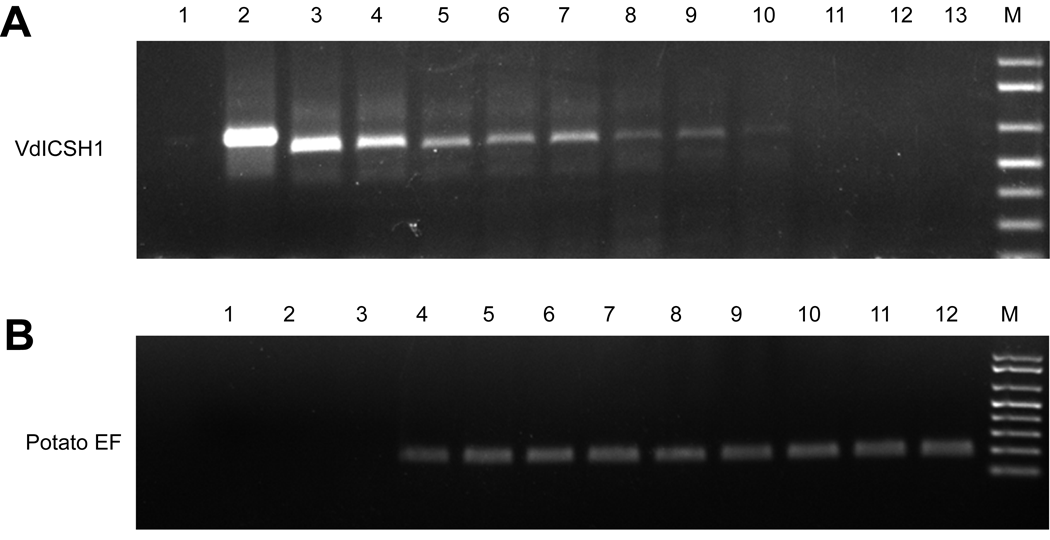

Supplement: FIGURE S1 — Amplification of V. dahliae ICSH1 in all potato detached leaves’ treatments at 1 DAI. Kennebec potato detached leaves from 4-week-old plants were placed in conidial suspensions of the V. dahliae highly aggressive isolate Vd1396-9, the weakly aggressive isolate Vs06-07, or sterilized distilled water as a control treatment. (A) Amplification of VdICSH1 in all potato detached leaves’ treatments at 1 DAI; lane 1: negative control; lane 2: Vd1396-9 genomic DNA; lane 3: cDNA of Vd1396-9; lane 4: cDNA of Vs06-07; lane 5–7: cDNA of potato detached leaves inoculated with Vd1396-9 at 1 DAI; lane 8–10: cDNA of potato detached leaves inoculated with Vs06-07 at 1 DAI; lane 11–13: cDNA of water control potato detached leaves at 1 DAI; M: DNA marker. (B) Amplification of potato elongation factor (EF) in all potato detached leaves’ treatments at 1 DAI; lane 1: negative control; lane 2: cDNA of Vd1396-9; lane 3: cDNA of Vs06-07; lane 4–6: cDNA of potato detached leaves inoculated with Vd1396-9 at 1 DAI; lane 7–9: cDNA of potato detached leaves inoculated with Vs06-07 at 1 DAI; lane 10–12: cDNA of water control potato detached leaves at 1 DAI; M: DNA marker. [file Image_1.TIF]

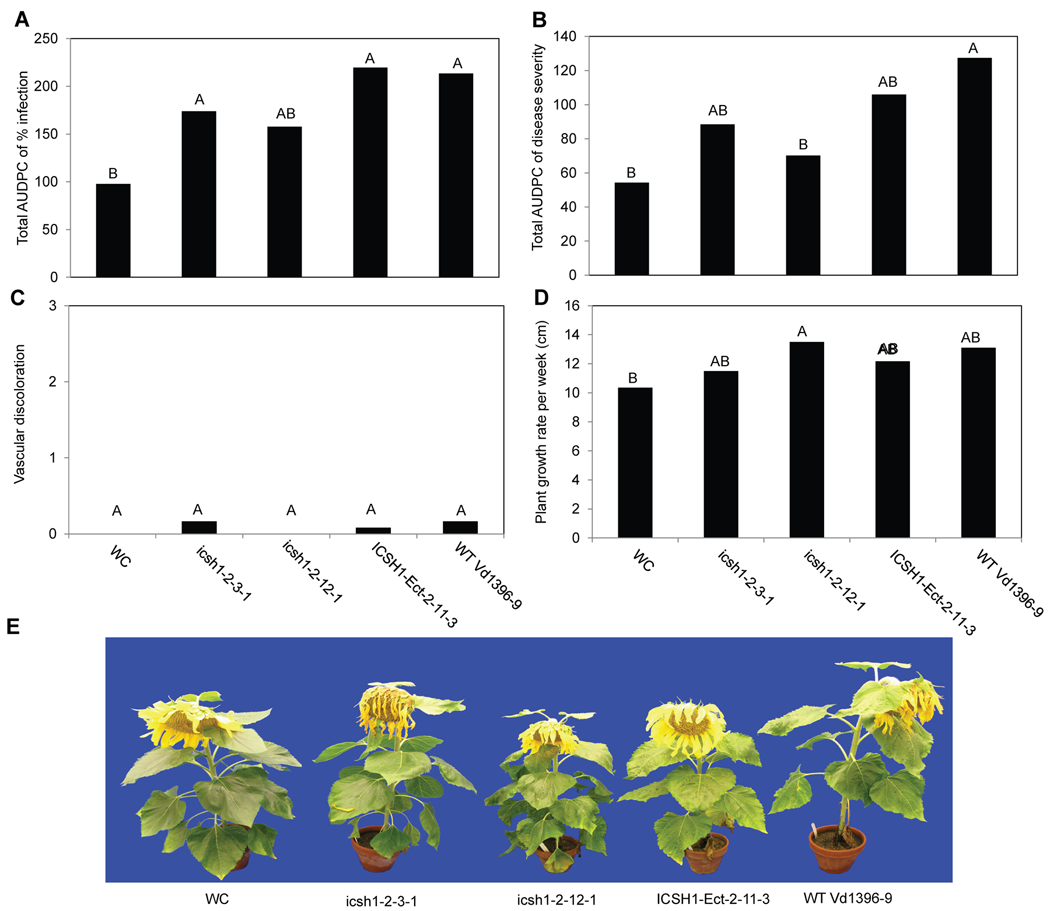

Supplement: FIGURE S2 — Pathogenicity of icsh1 mutant on susceptible sunflower line IS8048. Roots of the susceptible sunflower line IS8048 were washed and placed in a conidial suspension of V. dahliae. The percentage of infection, disease severity and plant height were recorded every week. The vascular discoloration of the stem cross-sections were rated at 8 weeks after inoculation, with icsh1-2-3-1 or icsh1-2-12-1; ectopic control: ICSH1-Ect-2-11-3; wild type: Vd1396-9; WC: water control. (A) Total AUDPC of percentage of infection; (B) Total AUDPC of disease severity; (C) Vascular discoloration; (D) Growth rate of susceptible sunflower; (E) Sunflower line IS8048 infected by icsh1 mutants at 8 weeks after infection. Bars represented by mean values (n = 4) sharing the same letter are not significantly different from each other (P < 0.05). This experiment was repeated twice. [file Image_2.TIF]

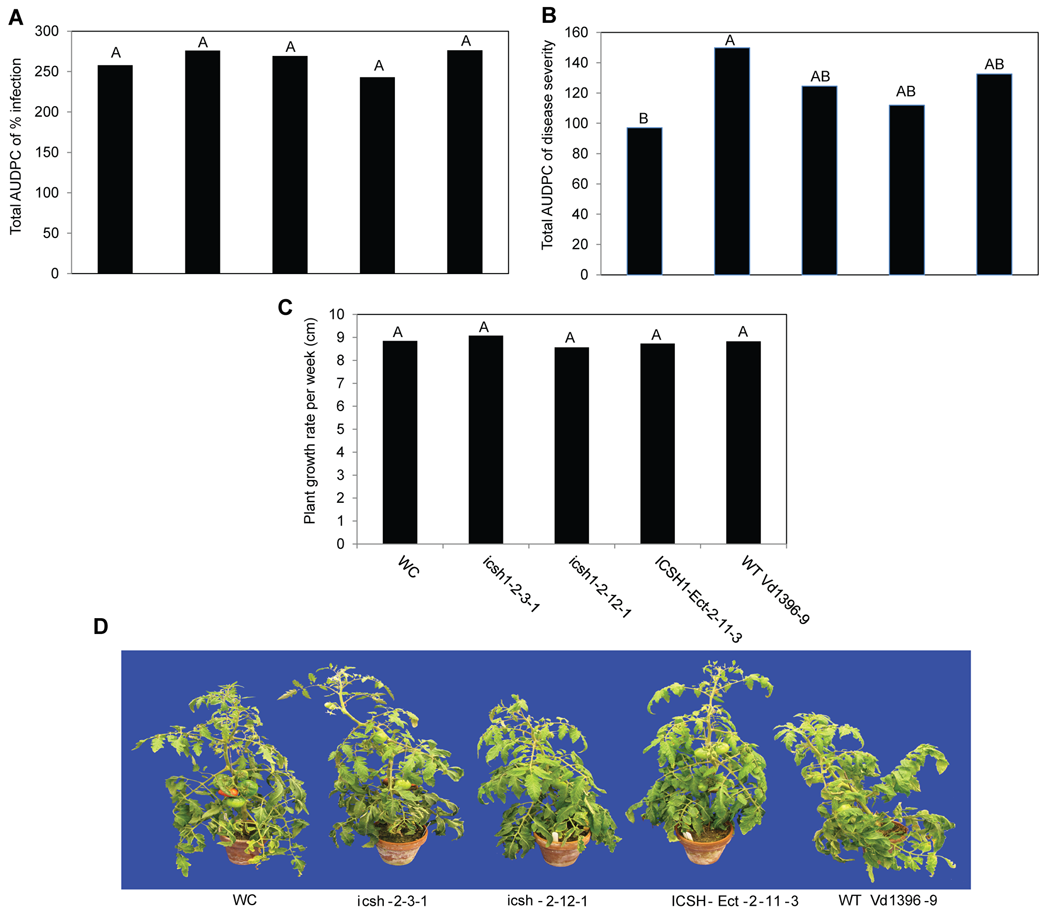

Supplement: FIGURE S3 — Pathogenicity of the icsh1 mutant on susceptible tomato variety Bonny Best. Roots of the susceptible tomato variety Bonny Best, were washed and placed in a conidial suspension of V. dahliae. The percentage of infection, disease severity and plant height were recorded for each week after inoculation with icsh1-2-3-1 or icsh1-2-12-1; ectopic control: ICSH1-Ect-2-11-3; wild type: Vd1396-9; WC: water control. (A) Total AUDPC of percentage of infection; (B) Total AUDPC of disease severity; (C) Growth rate of susceptible tomato; (D) Tomato variety Bonny Best infected by icsh1 mutants at 8 weeks after infection. Bars represented by mean values (n = 6) sharing the same letter are not significantly different from each other (P < 0.05). This experiment was repeated twice. [file Image_3.TIF]

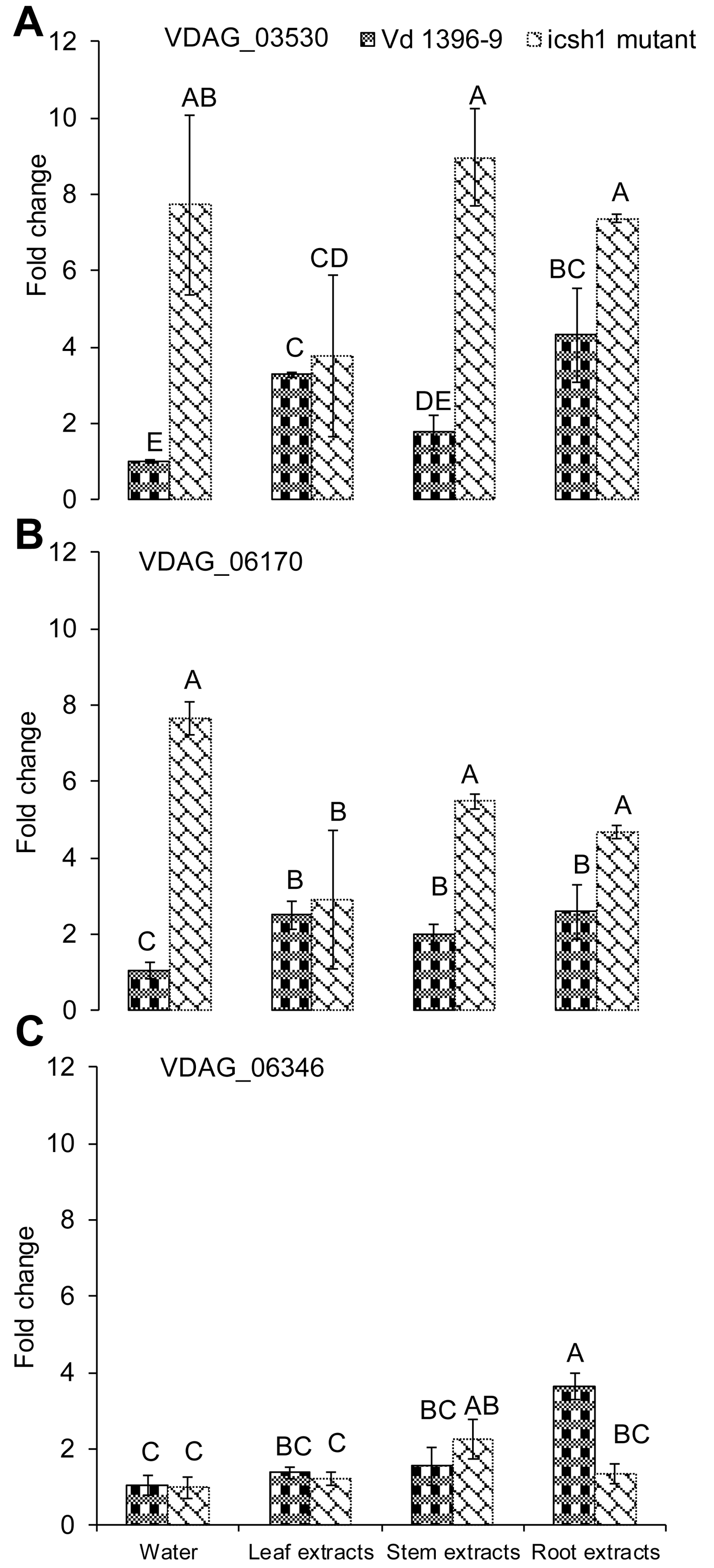

Supplement: FIGURE S4 — Expression of isochorismatase family members in the V. dahliae icsh1 mutant and wild type strain Vd1396-9 under elicitation with different potato tissue extracts. Verticillium dahliae wild type strain Vd1396-9 and icsh1-2-12-1 were induced in liquid media by different potato tissue extracts. Sterilized distilled water was used as a control treatment. QRT-PCR data was normalized using V. dahliae Histone H3. (A) VDAG_03530; (B) VDAG_06170; (C) VDAG_06346. Each gene’s expression data obtained from the icsh1 mutant and wild type strain Vd1396-9 under all treatments and control were analyzed using the 2-ΔΔCT method relative to that of wild type Vd1396-9 cultured in CDB medium with water. Bars represented by mean values (n = 3, with two technical replications) sharing the same letter are not significantly different from each other (P < 0.05). [file Image_4.TIF]
